# Supplementary material for: MetaRibo-Seq measures translation in microbiomes
Source: Nat Commun. 2020 Jun 29;11:3268. doi: 10.1038/s41467-020-17081-z (PMC7324362; doi:10.1038/s41467-020-17081-z)
Supplement: Supplementary file 10 — Supplementary Data 7 [file 41467_2020_17081_MOESM10_ESM.zip › File2/Confidence_High_Taxonomy/138573_out.krona.html]

Javascript must be enabled to view this page.

members
magnitude
magnitudeUnassigned
count
unassigned
taxon
rank

138573\_out

4

2
superkingdom
4

4
1239
phylum

4
186801
class

4
186802
order

family
541000
1

216851
genus
1

1
853

SRS146888\_contig\_number\_2871
species

3
family
216572

459786
genus
3

1945593

SRS019968\_contig\_number\_12075
species
1

1262911

SRS018656\_contig\_number\_5800SRS046369\_contig\_number\_10890
species
2
